# Respiratory Muscle Training in Mechanically Ventilated Adult Patients: Toward a Precise Prescription Based on Current Evidence: A Scoping Review

**DOI:** 10.3390/jcm14145058

**Published:** 2025-07-17

**Authors:** Jennifer Andrea Carabalí-Rivera, Valeria Salazar-Muñoz, Evelyn dayana Villanueva-Londoño, Katherine González-Ruiz, Leonardo Arzayus-Patiño

**Affiliations:** Physiotherapy Program, Faculty of Health, Universidad Santiago de Cali, Cali 760035, Colombia; jennifer.carabali00@usc.edu.co (J.A.C.-R.); valeria.salazar00@usc.edu.co (V.S.-M.); evelyn.villanueva00@usc.edu.co (E.d.V.-L.); katherine.gonzalez07@usc.edu.co (K.G.-R.)

**Keywords:** patients, breathing exercises, artificial respiration, ventilators, mechanical, muscle strength, respiratory muscles, intensive care units, adult, respiratory muscle training, hospital mortality

## Abstract

Respiratory muscle training (RMT) has been proposed as a supportive strategy for adults receiving invasive mechanical ventilation; however, the way RMT is prescribed—mode, intensity, frequency, and volume—remains highly heterogeneous. **Objectives**: This study aimed to describe the current evidence regarding the prescription of respiratory muscle strengthening in terms of frequency, intensity, method, and volume in adult patients under mechanical ventilation in intensive care units. **Methods**: A scoping review was conducted following the PRISMA-ScR guidelines based on searches in electronic databases including Scopus, SciELO, ScienceDirect, PubMed, LILACS, Springer, Web of Science, Google Scholar, PEDro, Dialnet, and Cochrane. **Results**: Seven studies met the established inclusion criteria and described prescription protocols for respiratory muscle strengthening in adult patients under mechanical ventilation in intensive care units. **Conclusions**: The most frequently reported protocol involved threshold load training at 40–50% of maximal inspiratory pressure, administered twice daily, every day of the week, with a volume of 30 repetitions. This intervention showed promising results in improving inspiratory muscle strength, with potential additional benefits in weaning success and pulmonary function.

## 1. Introduction

Prolonged stays in the intensive care unit (ICU) often lead to neuromuscular complications, as well as deterioration in physical function and mental health status [1]. Approximately 35% of ICU admissions require mechanical ventilation (MV) as part of life-support treatment for acute respiratory failure [2,3]. Among these patients, 20–30% experience difficulties during the weaning process, and nearly 50% require MV for seven days or more [2]. This can result in complications such as altered respiratory mechanics, airway injuries, acute lung injury, pneumonia, tracheal ischemia [3], and diaphragmatic weakness. Moreover, patients with weaning difficulties consume around 40% of ICU resources, significantly increasing the daily cost of care [2].

Mechanical ventilation alone can induce early atrophy of diaphragmatic muscle fibers. Evidence shows that within just 18 h of controlled ventilation, these fibers may decrease in size by up to 57%, significantly compromising their contractile strength. This diaphragmatic weakness has important clinical consequences, including secretion retention and an increased risk of pulmonary collapse [4,5], ultimately delaying weaning and extubation, and increasing mortality [6].

Extended ICU stays are associated with rapid loss of muscle mass, strength, and bone mineral density, particularly during the first week of bed rest. Generalized muscle weakness affects between 30% and 60% of critically ill patients and may persist for up to two years after hospital discharge [7]. This condition contributes to functional decline, diminished quality of life, prolonged MV duration, and impaired pulmonary function.

Respiratory muscle weakness is a common finding in critically ill patients [8], often presenting as diaphragmatic atrophy, with up to a 10% reduction in diaphragm thickness observed during the first few days of MV. This condition affects 63% to 80% of patients who remain on ventilatory support for more than 48 h and may persist even after ventilator withdrawal [2,6]. However, this weakness can be mitigated through respiratory muscle training (RMT), a strategy that has shown to improve diaphragmatic strength and endurance, thereby facilitating weaning and reducing complications [8].

RMT can be delivered through various strategies, all based on the physiological principle of applying a threshold load that opposes the negative or positive pressure generated by the respiratory muscles. One modality involves modifying ventilator settings to adjust trigger sensitivity, with training intensities ranging from 20% to 70% of the maximal inspiratory pressure (MIP) [9]. In addition, several devices have been developed, including flow-resistive loading devices, pressure-threshold devices, and voluntary isocapnic hyperpnea systems [10].

Among the most commonly used devices are the Philips Respironics Threshold IMT, which targets inspiratory muscle strengthening, and the Respironics Threshold PEP, which focuses on expiratory muscles [6,8]. The PFLEX Resistive Trainer imposes airflow resistance through a small-caliber orifice, increasing workload primarily during expiration. Meanwhile, the SpiroTiger enables simultaneous inspiratory and expiratory training by increasing respiratory rate and tidal volume [11]. These devices provide adjustable and consistent resistance, allowing for targeted and individualized respiratory muscle training, which is essential for improving muscular strength.

Although RMT has demonstrated clinical effectiveness, there remains substantial variability in the prescription protocols used across studies. Research differs in key parameters such as threshold load, session frequency, number of repetitions, and total training volume, making it difficult to establish a consensus on optimal prescription strategies. For healthcare professionals, understanding these parameters is crucial to optimize resource use, apply evidence-based interventions, and enhance clinical outcomes.

In this context, the present scoping review aims to describe the current evidence regarding the prescription of respiratory muscle training in adult patients undergoing mechanical ventilation in the ICU. Additionally, it seeks to identify the characteristics of the populations studied, the properties of the implemented intervention strategies, and the reported clinical outcomes. Accordingly, the following question is posed: What are the prescription parameters for respiratory muscle training in adult patients undergoing mechanical ventilation in the ICU?

## 2. Materials and Methods

### 2.1. Methods

This review followed the guidelines established by the PRISMA-ScR (Preferred Reporting Items for Systematic Reviews and Meta-Analyses extension for Scoping Reviews) checklist [12] and was based on the methodology developed by the Joanna Briggs Institute (JBI). These guidelines include the following phases: formulation of the research question, identification of relevant studies, study selection, data extraction, synthesis and presentation of results, and conclusions.

### 2.2. Research Question

The following research question was proposed to guide this review: What are the prescription parameters for respiratory muscle strengthening in adult patients undergoing mechanical ventilation in the intensive care unit?

The question was structured using the PICO format with the following components:

P (Population): Adult patients on mechanical ventilation (MV) in the ICU

I (Intervention): Respiratory muscle training (RMT)

C (Comparison): Not applicable

O (Outcomes):

Primary outcome: Prescription parameters (frequency, intensity, repetitions, volume, mode)

Secondary outcomes: Improvement in respiratory muscle strength, duration of MV, mortality, ICU length of stay, complications, and safety

### 2.3. Eligibility Criteria

The researchers conducted a literature search based on the proposed research question. Articles were selected according to the following inclusion criteria: randomized controlled trials (RCTs) and descriptive or analytical observational studies that addressed the PIO question. No restrictions were applied regarding the publication date, and studies were included if published in Spanish, English, or Portuguese.

Exclusion criteria:

Studies involving complementary interventions in addition to respiratory muscle training—such as pulmonary re-expansion techniques that could alter the outcomes—were excluded.

### 2.4. Information Sources

Based on the research question, a comprehensive search was conducted in multiple electronic databases, including Scopus, Scielo, ScienceDirect, PubMed, LILACS, Springer, Web of Science, Google Scholar, PEDro, Dialnet, and Cochrane. The search strategy incorporated both Medical Subject Headings (MeSH) and Descriptores en Ciencias de la Salud (DeCS). The primary search terms included the following:

“Patients” OR “Adult” OR “Respiration, Artificial” OR “Ventilators, Mechanical” OR “Intensive Care Units” AND “Breathing Exercises” OR “Respiratory Muscles” OR “Exercise Therapy” AND “Muscle Strength” [MeSH].

### 2.5. Search Strategy

Both controlled and uncontrolled vocabulary were used, with search terms derived from Medical Subject Headings (MeSH), Descriptores en Ciencias de la Salud (DeCS), and keywords organized according to the PICO framework in English, Spanish, and Portuguese. Specific search equations were designed for each database. Additionally, the search process and terminology were discussed and reviewed in collaboration with a search strategy specialist.

To improve search precision, filters were applied to reduce the number of irrelevant studies, increase comprehensiveness, and ensure maximum coverage.

The search equations are presented in Table 1. Duplicates were identified and removed. Subsequently, the search results from each database were distributed evenly among three independent reviewers, who screened, selected, and extracted data individually. Initially, the titles and abstracts of the identified articles were assessed, and those not meeting the inclusion criteria were excluded. The remaining full-text articles were then reviewed in detail to verify their eligibility based on the predefined criteria. Finally, studies that fulfilled all the inclusion requirements were incorporated into this review.

### 2.6. Selection of Sources of Evidence

The process of identification, screening, and eligibility assessment was carried out by consensus among the researchers. A fourth investigator served as a reviewer and was responsible for resolving any discrepancies.

### 2.7. Data Extraction

Once the articles were identified, the researchers conducted a critical reading and compiled a descriptive table with the relevant data from each study. One team member reviewed and analyzed the articles to determine their relevance and compliance with all inclusion criteria. No discrepancies were reported among the reviewers during this process.

Table 2 presents the main characteristics of the included studies: author, year, country, study objective, study design, evaluated variables, type of training or device used, prescription parameters, research findings, and conclusions.

### 2.8. Critical Appraisal

The methodological quality of the randomized controlled trials (RCTs) was assessed using the PEDro Scale, a validated tool composed of 11 items. Item 1 assesses external validity, items 2 through 9 evaluate internal validity, and items 10 and 11 refer to statistical reporting and interpretability of results. Total scores range from 0 to 10, as item 1 is not included in the final PEDro score [13]. A score of ≥6 is considered indicative of high methodological quality. The appraisal results are summarized in Table 3.

The quality of the retrospective cohort study was evaluated using the Joanna Briggs Institute (JBI) Critical Appraisal Checklist for Cohort Studies [14], which examines potential sources of bias in study design, conduct, and analysis. The checklist includes items on inclusion criteria, measurement of exposure and outcomes, handling of confounding factors, and completeness of follow-up. The total percentage of criteria met was calculated to reflect overall quality. The results are presented in Table 4.

All quality assessments were conducted independently by two trained reviewers. Discrepancies were resolved by consensus or consultation with a third reviewer when necessary.

**Table 2:** Description of the main characteristics of the studies.

Author/Year/Country	Study Objective	Sample/Groups	Study Design	Evaluated Variables	Type of Training/Devices	Training Prescription	Study Findings	Conclusion
Bernie M. Bissett et al. [8] 2023 Australia	To determine whether high-intensity inspiratory training using a threshold loading device improves inspiratory muscle strength, as well as quality of life, dyspnea, and physical function, in patients undergoing mechanical ventilation for 7 days or more.	70 participants: 33 in the IMT group and 37 in the control group	Randomized controlled clinical trial (RCT)	Maximal inspiratory pressure (MIP), fatigue resistance index (FRI), SF-36v2 and EQ-5D-3L, modified Borg scale, acute care index of function (ACIF)	High-intensity, low-repetition method/IMT Threshold HS730, Respironics NJ, USA	Intensity: 50% of MIP, progressively increased Frequency: 5 times/week, 5 sets of 6 breaths	There was no significant difference in inspiratory strength or endurance; however, improvements in quality of life were observed.	High-intensity IMT using a threshold device may improve dyspnea and quality of life, although it might not increase inspiratory muscle strength or endurance nor expedite ventilator weaning.
Barbara Kellerman Smith et al. [15] 2014 United States	To determine the response to threshold pressure load (TPL) in mechanically ventilated patients with weaning difficulties who underwent inspiratory muscle strength training (IMST).		Retrospective cohort study	SBT protocol, inspiratory load compensation (ILC) to determine its role in strengthening and weaning outcome, PImax	Threshold PEP (Philips Respironics, Murrysville, Pennsylvania), inverted to deliver an inspiratory threshold training load	Frequency: 5 times/week, up to 28 days. Sets of <1 min, 2 min rest between sets. Load: 5, 10, and 15 cmH_2_O	No significant differences between patients who failed versus those who succeeded in weaning.	Flow and volume measurements from inspiratory load compensation (ILC) may provide useful information about muscle capacity in patients with weaning difficulty, including their suitability for IMST. However, further research is needed to assess whether ILC has predictive value in the weaning process.
L.M. Sandoval Moreno et al. [2] 2019 Colombia	To evaluate the effectiveness of respiratory muscle training (RMT) on weaning and inspiratory muscle strength in patients undergoing mechanical ventilation for 48 h or more.	A total of 126 patients were randomized: 62 to the experimental group and 64 to the conventional group.	Randomized controlled trial with parallel groups, double-blind design	PImax, weaning from mechanical ventilation, weaning failure, need for non-invasive mechanical ventilation	Threshold IMT (Inspiratory Muscle Trainer: Threshold IMT; Respironics Inc., Murrysville, PA, USA)	Control group: Received standard ICU respiratory physiotherapy care, including respiratory therapy, physical therapy, and mechanical ventilation management Intervention group: Frequency: twice daily, 7 days a week. Protocol: 3 sets of 6 to 10 repetitions, with 2 min of rest between sets. Intensity: 50% of maximal inspiratory pressure (PImax). Volume: 14 inspiratory muscle training (IMT) sessions.	No significant differences were found in weaning time or need for non-invasive ventilation (NIV). However, 14 days of inspiratory muscle training (IMT) led to significant improvements in inspiratory muscle strength.	There were no statistically significant differences in the weaning period from mechanical ventilation or in the change in respiratory muscle strength between the experimental group and the control group. 66
Robledo L Condesa, et al. [16] 2013 Brazil	To evaluate the usefulness of inspiratory muscle training (IMT) in improving inspiratory muscle strength, tidal volume, and rapid shallow breathing index and in accelerating the weaning process from mechanical ventilation.	A total of 77 participants were randomized: 38 to the intervention group and 39 to the control group.	Randomized trial with concealed allocation	MIP, MEP, cardiorespiratory variables (HR, RR, MAP, SpO_2_), ventilator weaning, rapid shallow breathing index (RSBI)	Inspiratory threshold device	40% of PImax, 5 sets of 10 reps, 2 times/day, 6 days/week Control group: no IMT Both groups: passive or active-assisted limb mobilization, thoracic compression with rapid release at end-expiration, endotracheal suctioning, and positioning.	Increased PImax (+12 cmH_2_O), PEmax (95% CI: 2–44 cmH_2_O), and tidal volume (+73 mL); decreased RRBI (not significant) and weaning time (−3 days, not significant)	Inspiratory training during weaning improved maximal inspiratory and expiratory pressure, as well as tidal volume, with statistically significant differences compared to the control group. Although the rapid shallow breathing index improved more in the experimental group, the difference was not statistically significant.
Bruno da Silva Guimarães et al. [17] 2021 Brazil	To evaluate the effects of inspiratory muscle training (IMT) on weaning and survival in tracheostomized patients.	A total of 111 participants were included and randomized: 58 to the intervention group and 53 to the control group.	Prospective randomized controlled trial	PIMAX, timed inspiratory effort (TIE) index, ICU survival rate, Richmond Agitation-Sedation Scale, ventilator weaning.	POWER Breathe K-5 device (Technologies Ltd., Birmingham, United Kingdom)	2 sets of 30 breaths each. Subsets of 10 repetitions, with 3 min of rest. Intensity: 40% of MIP Control group: no protocol.	MIP increased: +18.5 cmH_2_O, TIE: 0.93 ± 0.73, and significant improvement in muscle strength (*p* = 0.001) Higher 60-day survival: 71.1% (intervention) vs. 48.9% (control), *p* = 0.030	The IMT program was associated with a significantly greater increase in absolute PImax values (26.1 ± 18.5 cm H_2_O) and the timed inspiratory effort (TIE) index (0.93 ± 0.73 cm H_2_O/s) compared to no intervention.
A Daniel Martin, et al. [18] 2011 United States	To determine whether an inspiratory muscle strength training (IMST) program improves extubation outcomes in patients with failed extubation (FTW).	A total of 69 patients were randomized, with 35 assigned to the IMST condition and 34 to the SHAM treatment.	Single-center, single-blinded, randomized controlled trial	Number of IMST and SHAM training sessions, maximum inspiratory pressure (MIP) before and after training, pressure generated at the tracheostomy tube during training, duration of progressively longer breathing trials (BT), weaning success rate, adverse events during IMST or SHAM treatments.	Threshold PEP, between −4 and −20 cmH_2_O. For the SHAM group, a Pflex device set to the largest opening was used.	Load: −4 to −20 cmH_2_O, 4 sets of 6–10 repetitions, once per day, 5 days per week, for 28 days. SHAM group: constant minimal load using the largest valve setting.	Increased PImax and inspiratory muscle strength; higher successful extubation rate: 71% (IMST) vs. 47% (SHAM). No significant adverse events were reported.	Improvement in PImax outcomes and weaning success with IMST compared to sham training in patients with failed weaning (FTW).
Farnoosh Khodabandelo, et al. [19] 2023 Iran	To determine the effect of inspiratory muscle training (IMT) on weaning duration in patients admitted to the ICU.	Seventy-nine ICU-admitted patients were randomly assigned to intervention (n = 40) and control (n = 39) groups.	Double-blind randomized clinical trial	Primary variables: duration of mechanical ventilation, weaning duration, weaning success, maximum inspiratory pressure (MIP), peak expiratory flow (PEF), rapid shallow breathing index (RSBI), and pulmonary compliance. Secondary variables: continuation of invasive ventilation via tracheostomy, continuation of mechanical ventilation, reintubation within 24 h, and mortality.	Inspiratory muscle training (IMT) with a threshold device, in combination with conventional physiotherapy for patients in the intervention group. The control group received only conventional physiotherapy.	Intensity: 50% of MIP, 5 sets of 6 repetitions, 1 min rest between sets. Frequency: daily until successful weaning. Control group: Conventional physiotherapy only (percussion, vibration, mobilization).	Shorter weaning duration: IMT group—8.4 days vs. control group—11.2 days. Increased MIP, pulmonary compliance, and weaning success rate (54%). Also observed an increase in respiratory muscle strength.	Inspiratory muscle training (IMT) with a threshold device significantly improved weaning time and success, maximal inspiratory pressure (MIP), and pulmonary compliance in ICU patients, increasing the likelihood of successful weaning by 54% compared to the control group.

Abbreviations: IMT = inspiratory muscle training, PImax/MIP = maximal inspiratory pressure, MEP/PEmax = maximal expiratory pressure, RSBI = rapid shallow breathing index, TIE = timed inspiratory effort, FRI = fatigue resistance index, EQ-5D-3L = EuroQol Five Dimensions Three Levels, SF-36v2 = Short Form 36 Health Survey version 2, ACIF = acute care index of function, ILC = inspiratory load compensation, SBT = spontaneous breathing trial, PEF = peak expiratory flow, SpO_2_ = oxygen saturation, HR = heart rate, RR = respiratory rate, MAP = mean arterial pressure, ICU = intensive care unit, NIV = non-invasive ventilation, FTW = failure to wean, SHAM = simulated training group.

**Table 3:** Quality assessment according to Pedro scale for controlled clinical trial type studies.

Studies	The Selection Criteria Were Specified	Subjects Were Randomly Assigned to Groups (in a Crossover Study, Subjects Were Randomized as They Received Treatments)	Allocation Was Hidden	The Groups were Similar at Baseline Regarding the Most Important Prognostic Indicators	All Subjects Were Blinded	All Physiotherapists Who Administered the Therapy Were Blinded	All Evaluators Who Measured at Least One Key Outcome Were Blinded	Measures of at Least One of the Key Outcomes Were Obtained from More Than 85% of the Subjects Initially Assigned to the Groups	Results Were Presented for All Subjects Who Received Treatment or Were Allocated to the Control Group, With at Least One Key Outcome Analyzed by “Intention to Treat”	Results of Statistical Comparisons Between Groups Were Reported for at Least One Key Outcome	The Study Provides Point Measures and Variability for at Least One Key Outcome	Total
Bernie M. Bissett et al. [8]	1	1	1	1	1	1	1	1	1	1	1	11/11
L.M. Sandoval Moreno et al. [2]	1	1	1	1	1	1	1	0	1	1	1	10/11
Robledo L Condesa et al. [16]	1	1	1	1	1	1	0	0	1	1	1	9/11
Bruno da Silva Guimarães et al. [17]	1	1	0	1	0	0	0	1	1	1	1	7/11
A Daniel Martin, Barbara K Smith et al. [18]	1	1	1	1	1	0	0	1	1	1	1	9/11
Farnoosh Khodabandeloo et al. [19]	1	1	1	1	1	0	1	1	1	1	1	10/11

1 meets the criteria. 0 does not meet the criteria.

**Table 4:** Quality assessment according to the JBI checklist for retrospective cohort studies.

Studies	Were the Two Groups Similar and Recruited from the Same Population?		Were the Exposures Measured Similarly to Assign People to Both Exposed and Unexposed Groups?		Was the Exposure Measured in a Valid and Reliable Way?		Were Confounding Factors Identified?		Were Strategies to Deal with Confounding Factors Stated?		Were the Groups/Participants Free of the Outcome at the Start of the Study (or at the Moment of Exposure)?		Were the Outcomes Measured in a Valid and Reliable Way?		Was the Follow-Up Time Reported and Sufficient to Be Long Enough for Outcomes to Occur?		Was Follow-Up Complete, and if Not, Were the Reasons for Loss to Follow-Up Described and Explored?		Were Strategies to Address Incomplete Follow-Up Utilized?		Was Appropriate Statistical Analysis Used?	
Barbara Kellerman Smith et al. [15] 2013 United States	Yes	No	Yes	No	Yes	No	Yes	No	Yes	No	Yes	No	Yes	No	Yes	No	Yes	No	Yes	No	Yes	No
	Unclear	Not applicable	Unclear	Not applicable	Unclear	Not applicable	Unclear	Not applicable	Unclear	Not applicable	Unclear	Not applicable	Unclear	Not applicable	Unclear	Not applicable	Unclear	Not applicable	Unclear	Not applicable	Unclear	Not applicable

### 2.9. Presentation of Results

**Figure 1:**
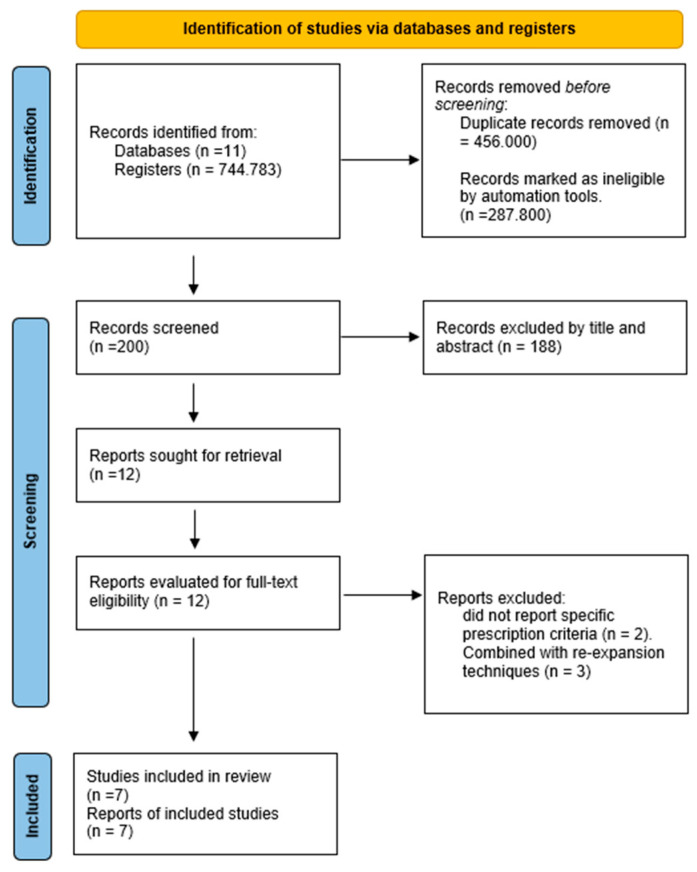
PRISMA flow diagram of the study selection process.

The results are presented in descriptive tables that highlight key aspects of each study, including the objective, population, sample size, methodological design, evaluated variables, type of training or device used, prescription parameters, findings, and conclusions. Additionally, tables are provided to illustrate the search strategy (Table 1), including the search equations used, and the final number of studies included. A flowchart (Figure 1) is also presented, detailing the study selection process and the final number of included studies. Supplementary Table S1 describes in detail the full-text articles that were excluded, along with the specific reasons for their exclusion.

## 3. Results

### 3.1. Literature Search

An initial total of 744,783 records were identified across eleven electronic databases. After duplicate removal and application of the predefined inclusion and exclusion criteria, the vast majority of records were excluded for not meeting the specific focus of this review. Most excluded studies either lacked a detailed description of respiratory muscle training (RMT) prescription parameters, involved non-relevant patient populations, or combined RMT with other interventions that could confound the outcomes.

Ultimately, only seven studies met all eligibility criteria and were included in the analysis (six randomized controlled trials and one retrospective cohort study). The marked difference between the initial number of records and the final number of included studies reflects both the high sensitivity of the search strategy and the limited availability of research focused exclusively on RMT prescription protocols in mechanically ventilated critically ill patients.

Table 2 presents the main characteristics of the analyzed studies, including author/year/country, study objective, sample/group, study design, evaluated variables, type of training/device, prescription parameters, research findings, and conclusions.

### 3.2. Methodological Quality Assessment

Of the six RCTs included, five were classified as having high methodological quality according to the PEDro scale [2,8,16,18,19], while one was considered of moderate quality [17]. The main methodological limitations identified were the lack of allocation concealment and participant blinding (criteria 3 and 5) in one study [17]; lack of therapist blinding (criterion 6) in three studies [17,18,19]; absence of assessor blinding (criterion 7) in three studies [16,17,18]; and insufficient follow-up completion (criterion 8) in two studies [2,16]. These limitations, although common in rehabilitation trials, may influence the internal validity of the findings. The results of this appraisal are detailed in Table 3.

The retrospective cohort study [15] was evaluated using the JBI Critical Appraisal Checklist and fulfilled 66% of the applicable items. Overall, 19% were not met and 15% were rated as unclear. Specific concerns included a lack of clarity in strategies to manage confounders (criterion 5), insufficient follow-up duration (criterion 8), inadequate statistical analysis (criterion 11), and failure to address loss to follow-up (criteria 9 and 10). The evaluation is summarized in Table 4.

The included studies were conducted in diverse settings, including Australia [8], the United States [15,18], Colombia [2], Brazil [16,17], and Iran [19], which may enhance the external applicability of findings across different healthcare systems.

### 3.3. Respiratory Muscle Training

The most commonly used protocol consisted of pressure-threshold training. Four of the studies [2,8,16,19] used the Threshold IMT device, while two studies [15,18] employed the Threshold PEP device in reverse to train the inspiratory muscles. Another study [17] utilized flow-resistive loading with the Power-Breathe device.

### 3.4. Training Prescription

The most common prescription using the Threshold IMT device in three studies [2,8,19] was an intensity of 50% of maximal inspiratory pressure (MIP), while one study [16] used an intensity of 40% of MIP. Regarding training volume, three studies [8,16,19] applied five sets of 6 to 10 breaths, with 1 to 2 min of rest between sets. One study [2] used three sets with the same number of repetitions and rest intervals.

In terms of frequency, two studies [2,16] implemented training twice daily, seven days per week; one study [8] prescribed it once daily, five days per week; and one study [19] administered training daily until successful weaning.

In the two studies using the Threshold PEP device (positive expiratory pressure) [15,18], the prescription included four sets of 6 to 10 breaths, with 2 min of rest between sets, and a frequency of once daily, five days per week. The intensity in one study [15] was 5, 10, and 15 cmH_2_O, while in the other [18], it ranged from −4 to −20 cmH_2_O.

The Power-Breathe device [17] was prescribed at an intensity of 40% of MIP, with a volume of two sets of 30 breaths and 2 to 3 min of rest between sets, performed once daily, five days per week.

### 3.5. Clinical Variables

Among the seven studies included, the most frequently evaluated variables—aside from respiratory muscle strength—were related to weaning from mechanical ventilation (MV) [2,15,16,17,18,19]. Three studies [2,18,19] specifically reported weaning failure and success rates. Two studies [8,19] assessed the duration of mechanical ventilation. Two studies [8,17] reported hospital mortality and ICU survival rates, while two other studies [16,19] included the rapid shallow breathing index (RSBI). One study [8] also evaluated the fatigue resistance index, quality of life, dyspnea, and physical function. One study [15] examined inspiratory load compensation, and another study [18] assessed breathing trials and adverse events during treatment.

## 4. Discussion

The primary objective of this study was to describe and present the available evidence regarding the most commonly used respiratory muscle training (RMT) protocols in adult patients undergoing mechanical ventilation in the intensive care unit (ICU). The findings indicate that the most frequently employed protocol involved pressure-threshold training, with prescriptions ranging from 5 to 7 days per week, one to two sessions per day, and a volume of five sets of 6 to 10 breaths [2,8,16,19].

In most of these studies [2,8,16,19], the training intensity ranged between 40% and 50% of the maximal inspiratory pressure (MIP). This parameter has been supported by studies such as that of Farnoosh Khodabandelo et al. [19], who reported that training at 50% of MIP was effective in reducing weaning time and increasing the success rate. However, it remains unclear whether progressive loading, as commonly implemented in peripheral muscle training, can yield greater improvements in respiratory muscle strength and endurance. This highlights the need for further research in this area.

Regarding the training prescription reported across studies, the most commonly used approach involved a frequency of 5 to 7 days per week, with one to two sessions per day and a volume of five sets of 6 to 10 breaths [2,8,16,19]. This suggests that both training frequency and volume are key factors in achieving effective outcomes. However, considerable variability exists among the prescriptions, underscoring the importance of individualizing the training protocol based on patient characteristics and response to treatment. Such variability likely reflects differences in patient populations and the specific objectives of each study.

Several studies have demonstrated that respiratory muscle training (RMT) significantly improves inspiratory muscle strength [15,19]. Notably, this intervention has proven effective in patients experiencing difficulty weaning from mechanical ventilation. These findings are consistent with those reported by Bruno da Silva Guimarães et al. [17], who showed that inspiratory muscle training (IMT) significantly enhanced inspiratory muscle strength and improved clinical outcomes, thereby facilitating ventilator weaning.

Secondary variables assessed across the studies suggest that IMT not only supports successful weaning but also enhances quality of life [8,17,19]. This may be attributed to increased inspiratory strength, which eases ventilator withdrawal and promotes greater patient independence following hospital discharge. Improved respiratory muscle function also contributes to better tolerance to fatigue and dyspnea. These results align with the study by Bernie M. Bissett et al. [8], which emphasized the importance of considering both inspiratory strength and improvements in quality of life and weaning time when evaluating critically ill patients.

Among the training devices, the Threshold IMT (pressure-threshold device) was the most commonly used across studies [2,8,16,19]. However, in study [17], a PowerBreathe device (flow-resistive loading) was employed. This suggests that respiratory muscle training should adhere to the principle of progressive loading based on a percentage of the patient’s maximal inspiratory pressure, in accordance with fundamental training principles. The widespread use of Threshold IMT is likely due to its ability to provide adjustable inspiratory loads and its user-friendly design, as supported by Farnoosh Khodabandelo et al. [19], who reported similar improvements in respiratory muscle strength using this device.

The Threshold PEP device was reported in two studies [15,18], where it was used in an inverted manner—delivering inspiratory threshold loading through the expiratory port. This represents a variation from its conventional application as described in the literature. Since the Threshold PEP provides a lower threshold resistance (5–20 cmH_2_O) than the Threshold IMT (9–41 cmH_2_O) [19], it was used inversely in patients who had difficulty triggering the valve on the Threshold IMT [18]. This adaptation illustrates the versatility of threshold loading devices to accommodate individual patient needs. Its reverse application may facilitate the initiation of training in patients with severe inspiratory muscle weakness. These findings highlight the importance of evaluating clinical responses to different training modalities and underscore the need for further research to validate the efficacy and safety of this alternative approach in clinical practice.

In terms of ventilator weaning, several studies [2,18,19] reported that IMT improved extubation success rates in patients with difficult weaning. This is likely because respiratory muscle strengthening is a key factor in the weaning process, particularly when prolonged weaning is primarily due to ICU-acquired muscle weakness rather than underlying pulmonary dysfunction. These findings are consistent with the study by Daniel Martin et al. [18], which demonstrated that inspiratory muscle strength training (IMST) is an effective strategy to enhance inspiratory strength and facilitate ventilator liberation in critically ill patients.

Weaning from mechanical ventilation is a complex, multifactorial process in which respiratory muscle weakness represents only one of several potential barriers. Our review intentionally refrains from implying causality between RMT and successful extubation. Instead, it aims to provide a detailed overview of how RMT is currently prescribed, enabling future trials to evaluate standardized protocols in patient subgroups where diaphragmatic weakness is the predominant limitation—rather than parenchymal, neurological, or hemodynamic factors. By clarifying key principles of dose, timing, and progression—elements often well established in peripheral muscle training—we aim to reduce one source of variability that currently obscures the true contribution of RMT within comprehensive weaning strategies.

Although the RMT protocols included in this review demonstrated considerable heterogeneity in terms of intensity, duration, frequency, and device type, certain patterns were observed. For example, most studies employed threshold loads of 40–50% of maximal inspiratory pressure (PIMax), with sessions conducted once or twice daily—suggesting an emerging consistency in prescription practices. However, methodological differences across studies limit the direct comparison of outcomes and emphasize the need to standardize key components of RMT protocols. This variability may also account for the discrepancies observed in the magnitude of benefits reported, particularly for secondary outcomes such as quality of life and weaning success.

One of the main limitations identified across the included studies is the marked clinical heterogeneity in respiratory muscle training protocols. This variability is evident in the type of device used (e.g., Threshold IMT, POWERbreathe, flow-resistive exercises, inverted PEP), the prescribed training intensity (ranging from submaximal loads of 30% to controlled progressions reaching 60–80% of maximal inspiratory pressure [MIP]), the frequency of sessions (ranging from 3 to 7 days per week), and the total volume of repetitions (from 15 to 60 per session). This methodological diversity directly impacts the comparability and interpretation of clinical outcomes.

In particular, studies employing higher and progressive loads, as well as devices with measurable load control, tended to report greater improvements in parameters such as maximal inspiratory pressure (MIP), tidal volume, and exercise tolerance when compared to interventions using non-load-specific devices such as flow-resistive trainers or lower-intensity protocols. For example, interventions that reached training intensities between 50–60% of MIP led to significant increases in inspiratory muscle strength (exceeding 20%) and sustained functional gains, in contrast to those with fixed, lower-intensity loads (30–40%). Similarly, protocols with higher frequency (5–7 days per week) were associated with a faster recovery of respiratory muscle strength, whereas those with lower frequency yielded more modest outcomes.

Moreover, contextual factors such as the variable length of hospital stay affected the total number of sessions received by participants, leading to unequal exposure to the intervention. This further contributed to the heterogeneity in treatment effects, complicating the ability to draw robust and generalizable conclusions.

Consequently, these methodological differences limit the potential to establish standardized recommendations for the optimal prescription of respiratory muscle training. This underscores the urgent need for standardization of intervention protocols, including consistent criteria for device selection, load control, minimum number of sessions, and core prescription parameters (intensity, frequency, and volume). Such standardization would enhance the comparability of study findings, improve the clinical applicability of respiratory training interventions, and support their integration into structured respiratory rehabilitation programs in both inpatient and outpatient settings.

The methodological evaluation of the included studies revealed that most randomized controlled trials (RCTs) demonstrated high methodological quality. Specifically, five RCTs achieved high scores on the PEDro scale [2,8,16,18,19], while one was rated as moderate in quality [17]. These findings suggest that the majority of the included trials adhered to robust methodological standards, supporting the internal validity and clinical applicability of their conclusions, particularly in guiding decision-making regarding respiratory muscle strengthening protocols.

In contrast, the retrospective cohort study [15] met only 66% of the criteria outlined in the Joanna Briggs Institute (JBI) critical appraisal tool. Notable limitations included inadequate strategies for addressing confounding variables, insufficient follow-up duration, and suboptimal statistical analysis. These methodological weaknesses may reduce the reliability of the findings and should be considered when interpreting the contribution of this study to the overall evidence. Nonetheless, its inclusion highlights the scarcity of non-randomized studies that specifically address respiratory muscle training prescription parameters in critically ill patients.

The strengths of this review lie in its ability to synthesize the available evidence on a highly relevant topic in the care of critically ill patients, providing a clearer understanding of the impact of respiratory muscle training. In addition, secondary outcomes—such as quality of life and weaning success—closely associated with inspiratory muscle strength, were considered, offering a more comprehensive view of the benefits of this intervention. In the field of physical therapy, these benefits include improved pulmonary function, reduced time on mechanical ventilation, and enhanced functional recovery in ICU patients, highlighting the fundamental role of physiotherapists in delivering evidence-based rehabilitation programs.

This review is also distinguished by the methodological rigor applied throughout its development, including adherence to established guidelines for scoping reviews and critical appraisal of study quality, thereby reinforcing the validity and clinical relevance of its findings.

However, this review has some limitations, including a lack of studies with long-term follow-up and limited diversity in patient populations, which restrict broader understanding of the sustained effects of respiratory muscle training. Moreover, heterogeneity in training prescriptions across studies made it difficult to directly compare and standardize regimens.

Therefore, it is recommended that future research include randomized controlled trials with larger sample sizes and varied training prescriptions, aiming to establish a standardized and evidence-based protocol for respiratory muscle training, similar to those employed in peripheral muscle rehabilitation.

## 5. Conclusions

Respiratory muscle strengthening, using a prescription based on a threshold load of 40–50% of the patient’s maximal inspiratory pressure, administered twice daily, every day of the week, and with a volume of 30 repetitions, has shown a consistent association with improvements in inspiratory muscle strength. While some studies have reported potential benefits for weaning, a reduction in complications, and enhanced pulmonary function, these outcomes should be interpreted with caution due to the multifactorial nature of the weaning process and variability in study designs. Further research using standardized training protocols is needed to better understand the extent of these effects.

## Figures and Tables

**Table 1:** Study search.

Database	ST	SA	FR	FS	Search Terms/Equation	Filters Applied
PubMed	442	60	20	2	(“Respiratory Muscles” [MeSH] AND “Breathing Exercises” [MeSH]) AND (“Ventilators, Mechanical” [MeSH]) AND (“Intensive Care Units” [MeSH])	Language: English, Spanish, Portuguese; humans
Scopus	270	40	10	1	TITLE-ABS-KEY(“Respiratory Muscle Training” AND “Mechanical Ventilation” AND “ICU”)	Language: English; research articles
SciELO	81	15	6	0	(“Entrenamiento muscular respiratorio” AND “ventilación mecánica”)	Language: Spanish; humans
LILACS	149	18	8	1	(“Entrenamiento muscular respiratorio” AND “ventilación mecánica” AND “unidad de cuidados intensivos”)	Language: Spanish and Portuguese; humans
ScienceDirect	1056	25	10	1	“Respiratory muscle training” AND “mechanical ventilation” AND “ICU”	Language: English; research article
PEDro	804	30	5	0	“Breathing exercise” AND “mechanical ventilation”	Clinical trials only
Dialnet	1705	5	2	0	(“Ejercicio respiratorio” AND “ventilación mecánica” AND “UCI”)	Language: Spanish; academic articles
SpringerLink	321	20	6	1	“Respiratory muscle training” AND “mechanical ventilation” AND “intensive care”	Language: English; human studies
Cochrane	4	2	2	0	“Breathing exercises” AND “mechanical ventilation”	Trials; humans; no date limit
Google Scholar	15800	5	3	0	“Respiratory muscle training” AND “mechanical ventilation” AND “intensive care unit”	Language: English/Spanish; manual filtering
Web of Science	720056	100	8	1	TS=(“Respiratory muscle training” AND “mechanical ventilation” AND “ICU”)	Language: English; humans; research articles

Abbreviations: ST: total studies found; SA: selected by abstract; FR: full text reviewed; FS: final studies included in the review.

## Data Availability

The original contributions presented in this study are included in the article/Supplementary Material. Further inquiries can be directed to the corresponding author(s).

## Supplementary Materials

The following supporting information can be downloaded at: https://www.mdpi.com/article/10.3390/jcm14145058/s1, Table S1: Studies evaluated in full text but excluded (n = 5) [6,20,21,22,23].
