# Supplementary material for: Respiratory Muscle Training in Mechanically Ventilated Adult Patients: Toward a Precise Prescription Based on Current Evidence: A Scoping Review
Source: J Clin Med. 2025 Jul 17;14(14):5058. doi: 10.3390/jcm14145058 (PMC12295891; doi:10.3390/jcm14145058)
Supplement: Supplementary file 1 [file jcm-14-05058-s001.zip › jcm-3714586-supplementary.pdf]

Suggested text for the supplementary material:

This supplementary material provides a comprehensive overview of the full-text articles excluded during the eligibility assessment phase of this scoping review. The reasons for exclusion were grouped into two primary methodological issues: (1) studies that implemented combined interventions—such as respiratory muscle training alongside pulmonary re-expansion techniques, general physiotherapy, or broader rehabilitation protocols—thereby preventing the isolation of the specific effects attributable to respiratory muscle training; and (2) studies that did not clearly report essential prescription parameters, such as training intensity, frequency, volume, or method, which limited the interpretability, reproducibility, and clinical applicability of the findings. The inclusion of this table aims to enhance the transparency of the study selection process and improve the reproducibility of the review, in alignment with best-practice recommendations for scoping reviews.

Table S1. Studies evaluated in full text but excluded (n = 73)

**Author (Year)**	**Journal**	Title	Reason for Exclusion
Weiner et al. (1997)[20]	Journal of Thoracic and Cardiovascular Surgery	The effect of incentive spirometry and inspiratory muscle training on pulmonary function after lung resection	Combined intervention (inspiratory muscle training + incentive spirometry)
Jenkins et al. (2023) [6]	Intensive Care Medicine Experimental	The metabolic cost of inspiratory muscle training in mechanically ventilated patients in critical care	Reported only the training load (4 cmH_2O, 30–50–80% of NIF), without specifying other key prescription parameters such as frequency, session volume, or duration.
Bissett et al. (2020) [21]	Aust Crit Care	Inspiratory muscle training for ICU patients: practical guide	Did not provide explicit details on prescription parameters (e.g., frequency, intensity, session volume), limiting reproducibility and standardization of the intervention.
Front Med RCT (2024) [22]	Frontiers in Medicine	Inspiratory muscle training facilitates	Although IMT was applied, the intervention

		liberation from mechanical ventilation in critically ill patients: a randomized trial	was implemented in conjunction with routine ICU care, confounding the isolated effect of respiratory muscle training.
Tambunan et al. (2013) [23]	Indo J Phys Med Re hab	Effect of Inspiratory Muscle Training with Incentive Spirometry to Improve Respiratory Capacity in COPD Patients	Combined intervention: IMT + incentive spirometry + breathing-control exercises, preventing isolation of IMT effect

.
